# Genome-wide identification of *terpenoid synthase* family genes in *Gossypium hirsutum* and functional dissection of its subfamily *cadinene synthase A* in gossypol synthesis

**DOI:** 10.3389/fpls.2023.1162237

**Published:** 2023-04-26

**Authors:** Tianyang Wen, Xiao Xu, Aiping Ren, Ge Zhao, Jiahe Wu

**Affiliations:** ^1^ Zhengzhou Research Base, State Key Laboratory of Cotton Biology, School of Agricultural Sciences, Zhengzhou University, Zhengzhou, China; ^2^ State Key Laboratory of Plant Genomics, Institute of Microbiology Research, Chinese Academy of Sciences, Beijing, China

**Keywords:** gossypol, phylogenetic analysis, cadinene synthase, *Gossypium hirsutum*, terpenoid synthase

## Abstract

Plant *terpenoid synthase (TPS)* family genes participate in metabolite synthesis, hormones, gossypol, etc. Here, we genome-widely identified *TPS* family genes in 12 land plant species. Four hundred and thirty TPS-related genes were divided into seven subfamilies. The *TPS-c* in Bryophytes was suggested to be the earliest subfamily, followed by the *TPS-e/f* and *TPS-h* presence in ferns. *TPS-a*, the largest number of genes, was derived from monocotyledonous and dicotyledonous plants. Collinearity analysis showed that 38 out of the 76 *TPS* genes in *G. hirsutum* were collinear within *G. arboreum* and *G. raimondii*. Twenty-one *GhTPS-a* genes belong to the cadinene synthase (*GhCDN)* subfamily and were divided into five groups, A, B, C, D, and E. The special *cis*-elements in the promoters of 12 *GhCDN-A* genes suggested that the JA and ethylene signaling pathways may be involved in their expression regulation. When 12 *GhCDN-A* genes were simultaneously silenced through virus-induced gene silencing, the glandular color of *GhCDN-A*-silenced plants was lighter than that of the control, supported by a gossypol content decrease based on HPLC testing, suggesting that *GhCDN-A* subgroup genes participate in gossypol synthesis. According to RNA-seq analysis, gossypol synthesis-related genes and disease-resistant genes in the glandular variety exhibited upregulated expression compared to the glandless variety, whereas hormone signaling-related genes were downregulated. All in all, these results revealed plant *TPS* gene evolution rules and dissected the *TPS* subfamily, *GhCDN-A*, function in gossypol synthesis in cotton.

## Introduction

When plants are attacked, a large amount of volatile terpenoids is released. Terpenoids are a class of natural products with extremely rich structures. More than 80,000 kinds of natural terpenoids and derivatives have been found in bacteria, fungi, insects, and plants (Yamada et al., 2012; Schmidt-Dannert, 2015; Tholl, 2015; Beran et al., 2016; Christianson, 2017). Terpenoids are the most abundant type of metabolites synthesized by plants and can be divided into primary metabolites and secondary metabolites in different plants according to their physiological functions. A small number of terpenoids play physiological roles as primary metabolites required for plant growth and development, such as gibberellin, abscisic acid, carotenoids, and chlorophyll (Tetali, 2019). Most terpenoids are secondary metabolites of plants and play an important role in life processes such as plant adaptation to the environment, transmission of information, and chemical defense. For example, some volatile terpenoids are the main substances produced by floral fragrance, which can attract pollinators. Some terpenoids can be used as plant toxins to defend against herbivores, pest insects, and pathogenic microorganisms. Some terpenoids also have ecological functions that mediate the interaction between plants and surrounding biotic and abiotic factors (Keeling and Bohlmann, 2006; Vaughan et al., 2013; Yang et al., 2013).

The formation of structural diversity in terpenoids mainly depends on terpenoid synthase (TPS) (Chen et al., 2011), which is a key enzyme in the synthesis of terpenoids in secondary metabolism. Almost all plants contain *TPS* genes (Chen et al., 2011). Currently, multiple *TPS* families have been identified in many species, such as *Arabidopsis thaliana*, *Vitis vinifera*, and *Glycine max* (Aubourg et al., 2002; Martin et al., 2010; Falara et al., 2011; Nieuwenhuizen et al., 2013; Chen et al., 2014; Irmisch et al., 2014; Liu et al., 2014; Keilwagen et al., 2017; Yu et al., 2020; Zhou et al., 2020). The TPS enzyme in plants can transform GPP (geranyl diphosphate), NPP (neryl diphosphate), FPP (farnesyl diphosphate), and GGPP (geranylgeranyl diphosphate), which are synthesized from IPP (isopentenyl diphosphate) and DMAPP (dimethylallyl diphosphate), which are generated by the MVA (mevalonate) or MEP (methylerythritol phosphate) pathway, into multiple sesquiterpenes, monoterpenes, and diterpenes (Lichtenthaler and Hartmut, 1999; Takahashi and Koyama, 2006; Sapir-Mir et al., 2008).

Gossypol is a sesquiterpene polyphenolic substance that can combine with proteins and phospholipids in cells of the body and affect normal body functions, so it has great toxicity (Qian and Wang, 1984). The synthesis of gossypol begins with acetyl-CoA from the MVA pathway in plants. FPS (farnesyl diphosphate synthase) catalyzes IPP to produce FPP (Liu et al., 2000), and cadinene synthase (CDN) catalyzes the formation of cadinene (CAD) from FPP (Chen et al., 1995; Meng et al., 1999; Tan et al., 2000). Then cadinene-7-hydroxylase catalyzes CAD and NADPH to produce 7-hydroxycadinene (Luo et al., 2010), which undergoes a series of reactions to form hemi-gossypol. The hemi-gossypol is modified to form the sesquiterpenoid gossypol (Zhao et al., 2019). In addition to the above enzymes, the cytochrome P450 enzyme, DH1 (dehydrogenase), and 2-odd-1 ferric (II)-dependent dioxygenase also take part in the regulation of gossypol synthesis (Tian et al., 2018).

CDN is the first step and the most critical rate-limiting step in the synthesis of gossypol. Previous reports showed that a large reduction in the content of gossypol can be detected in CDN-silencing diploid cotton plants, *Gossypium arboreum* and *G. raimondii*. However, in tetraploid cotton, *G. hirsutum*, some reports showed that silencing some *CDN* genes did not cause a large decrease in the content of gossypol, while their overexpression could increase the content of gossypol. Therefore, it was speculated that gene redundancy may be caused by other *CDN* subfamily members in tetraploid cotton (Chen et al., 1995; Davis et al., 1996; Xiao-Ya et al., 1996; Meng et al., 1999; Tan et al., 2000; Townsend et al., 2005).

The literature shows that the *CDN* family belongs to a large gene family and is mainly divided into four subfamilies: A, B, C, and D (Sun et al., 2010; Cheng et al., 2016). *CDN-A* and C subfamilies have a larger number of genes, which have a higher degree of sequence similarity, but their expression patterns are different: *CDN-A* is mainly expressed in seeds and roots, whereas *CDN-C* is mainly expressed in various tissues of cotton. Conversely, there are few studies on the members of the *CDN-B* and D subfamilies, suggesting that most of the members of these two subfamilies may be pseudogenes (Sun et al., 2010). Recently, the literature has documented that the *CDN* gene family can also be divided into five subfamilies, including A, B, C, D, and E, according to evolutionary time (Liu et al., 2015). At present, the classification and naming of the *CDN* gene family in cotton are confusing, which remains a completely and systematically explored study.

Cotton is an important cash crop in the world, producing natural fiber and oil, and its fiber plays an important role in the textile industry. Cotton production is limited by biotic stress, including pest insect and pathogen infection. This biotic stress in turn is partially overcome by the accumulated large amounts of gossypol and its related sesquiterpene aldehydes distributed in the epidermis of roots and the glands of the aerial part of the tissues (Coyle et al., 1994; Jan et al., 2000; Coutinho, 2002; Liu et al., 2002; Puckhaber et al., 2002; Lopez et al., 2005; Ye et al., 2005; Stipanovic et al., 2006; Wolter et al., 2006; Kline et al., 2008). A number of reports document that these secondary metabolites act as phytochemicals to protect cotton against pathogen infection and animal feeding (Cai et al., 2010). Additionally, gossypol in cottonseed is harmful to humans and animals (Paré and Tumlinson, 1997; Nagegowda, 2010; Tholl, 2015), because consumption of cottonseed oil or cake containing gossypol can lead to serious damage to the heart, liver, kidney, and reproductive system.

Given that TPS is a direct catalyst for the synthesis of terpenoids, with the in-depth exploration of the efficacy of terpenoids and the continuous development of molecular biological methods, the functional dissection of *TPS* genes has become a hotspot in plant terpenoid metabolic engineering. In this study, we focused on integrating genome-wide data and proteomic data to perform the genome-wide analysis of the terpenoid synthase genes in cotton through a combination of bioinformatic and molecular biology methods, involving the identification of the *TPS* gene family, conserved motif analysis, and tissue expression pattern. More importantly, we conducted evolution analysis on the *CDN* branch family of rate-limiting enzymes in gossypol synthesis and screened out the key genes to determine their function in gossypol synthesis through gene silencing, tissue expression analysis, and other experiments. The results shed light on the molecular mechanism of cotton terpenoid biosynthesis, which helps generate special cotton germplasm with no or less gossypol in seed.

## Materials and methods

### Plant material and treatment

*Gossypium hirsutum L*. cv. ‘CCRI24’ were grown in mixed soil under glasshouse conditions (14 h light (28–30 °C)/10 h dark (25–28 °C), 150 μmol/m^2^ s). To analyze organ- and tissue-specific gene-expression patterns in ‘CCRI24,’ plants were grown under field conditions at the Cotton Institute of the Chinese Academy of Agricultural Sciences, Zhengzhou, Henan, China. Tissues including root, stem, leaf, petal, stamen, pistil, calycle, ovules, and fiber at 5, 10, 15, 20, and 25 d were harvested for RNA extraction. To determine expression patterns, seedlings were grown in Hoagland’s solution for about 3 weeks, and 50 μM MeJA and 50 μM ethephon were added to Hoagland’s solution. The 2.5% alcohol solution and H_2_O were added to Hoagland’s solution as the corresponding mocks. Root, stem, and leaf samples were collected at 0, 0.5, 1, 3, 6, 12, 24, and 48 h, respectively, after treatment. Three samples were collected from each treatment, and each treatment included at least three biological replicates. All collected samples were immediately frozen in liquid nitrogen and stored at −80 °C for RNA or DNA extraction and subsequent analysis.

### Multiple sequence alignment and phylogenetic tree construction of *TPS* family genes

The website (https://phytozome-next.jgi.doe.gov/) was used to download the protein databases of 12 species, including *G. hirsutum*, *G. arboreum*, *G. barbadense*, *G. raimondii*, *Zea mays*, *Picea asperata*, *A. thaliana*, *Theobroma cacao*, *Oryza sativa*, *Populus trichocarpa*, *Sphagnum recurvum*, and *Selaginella moellendorffii*. The *TPS* gene family has the conserved PF01397 and PF03936 motifs, based on the published paper (Liu et al., 2014; Zhou et al., 2020; Jia et al., 2022). To identify all members of the *TPS* family genes, the PF01397 and PF03936 seed files were downloaded from the PFAM database (http://pfam.xfam.org/). The hidden Markov models of PF01397 and PF03936 were constructed with HMMER 3.0 (http://hmmer.org/). The sequences containing the protein motifs PF01397 and PF03936 were screened, and the obtained protein sequences were further verified by PFAM and the SMART database (https://smart.embl-heidelberg.de/). After manual correction, non-full-length sequences were removed to obtain candidate *TPS* family genes with a full-length open reading frame (ORF). Multi-sequence alignment of the *TPS* gene family within 12 species was performed with MEGA 7 with default parameters. An unrooted neighbor-joining as well as minimum-evolution tree was constructed with the P-distance model method and 1,000 bootstrap replications using the same alignment file. EvolView (https://evolgenius.info//evolview-v2) was used to beautify the phylogenetic tree.

### Chromosomal localization and collinearity analysis of *TPS* family genes

TBtools was used to extract the location information and chromosome length information of *TPS* family genes from the *G. hirsutum* genome. MapChart 1.0 was used to construct the visual map. One Step MCScanX in TBtools was used to complete the collinearity analysis in *G. hirsutum*, *G. arboretum*, and *G. raimondii*. The sequences with sequence similarity ≥70% among *TPS* family genes were screened out to calculate the d_N_/d_S_ values by the simple Ka/Ks calculator in TBtools.

### Identification of *TPS* family genes in *G. hirsutum*

ProtParam (https://web.expasy.org/protparam/) predicted molecular weight, isoelectric point, average hydrophilicity, and instability index of *GhTPS* family genes. The Plant-mPLoc website (http://www.csbio.sjtu.edu.cn/bioinf/plant-multi/) was used to predict the subcellular localization of the GhTPS protein family.

### Conserved motifs and gene structure analysis of *TPS-a* subfamily genes in *G. hirsutum*

The conserved motif of *TPS-a* subfamily genes in *G. hirsutum* was analyzed using the MEME website (https://meme-suite.org/meme/). Gene structure analysis was analyzed by the GSDS database (http://gsds.gao-lab.org/). TBtools was used to visualize output files from MEME and GSDS. The *TPS-a* subfamily genes of *G. hirsutum* were visualized in Excel, and then the motifs were marked and beautified in Adobe Illustrator.

### Promoter analysis of *CDN* subfamily genes

TBtools was used to extract a 2,500-bp sequence upstream of the *CDN-A* subfamily gene sequence as the promoter sequence. The Cotton FGD website (https://cottonfgd.net/) was used to confirm that it was indeed the promoter sequence of *CDN* subfamily genes and not the sequence of other genes. The PlantCARE website (http://bioinformatics.psb.ugent.be/webtools/plantcare/html/) was used for promoter element analysis, and TBtools was used to visualize the output image.

### VIGS (virus-induced gene silencing)

To obtain all gene silencing strains of the *GhCDN-A* subfamily, three conserved fragments were screened out except the conserved RR(P)X_8_W, RXR, DDXXD, and NSE/DTE motifs. A fragment of 534 bp was obtained by overlapping PCR, and *TRV::GhCDN-A* was constructed by VIGS. *Agrobacterium* containing recombinant plasmids was cultured in resistant medium containing kanamycin (50 mg/ml) and rifampicin (50 mg/ml) at 28 °C for one day (OD_600_ = 1.5–1.8). *Agrobacterium* was collected and re-suspended in the resuspension solution (10 mM MgCl_2_, 10 mM MES, and 200 μM acetosyringone) to an OD_600_ = 1.2. *TRV::GhCDN-A*, *TRV::00*, and *TRV::PDS* were mixed with the helper plasmid *TRV::192* in a ratio of 1:1. Cotton leaves with completely flat cotyledons but without true leaves were selected for injection (about 7 days after planting). The injected cotton was treated in a dark environment for 24 h and transferred to a normal environment for 10–14 days until the bleaching phenotype appeared. The leaves of *TRV::GhCDN-A* were used for silencing efficiency detection, and *TRV::00* was used as a negative control. Plants with silencing efficiencies below 50% were screened out for phenotype observation and photographed with cameras. At the same time, the content of gossypol was determined by 0.5 g of leaf tissue.

### Determination of gossypol content

Approximately 0.1 g of plant leaf was added to 1 ml of 70% acetone homogenate, extracted by ultrasound for 60 min, centrifuged at 8,000*g* for 10 min, and the supernatant was taken and filtered through a pinhead filter to be measured. Then the content of gossypol was determined by HPLC. HPLC liquid conditions: Rigol L3000 high-performance liquid chromatography; Sepax-C18 reverse phase column (250 mm ∗ 4.6 mm, 5 μm). The mobile phase preparation: A was 1% phosphoric acid aqueous solution, and the preparation method was to take 150 ml of filtered, ultra-pure water, add 1.5 mL of H_3_PO_4_, and mix it well. B was methanol. A: B = 15: 85. The sample size was 10 μl, the flow rate was 1.0 ml/min, the column temperature was 30 °C, the sample removal time was 40 min, and the UV wavelength was 235 nm.

### RNA-seq and data analysis

The material plants CCRI24 with and without gossypol were planted under culture conditions based on the Hoagland solution (Tocquin et al., 2003) with minor modifications. Cultures were kept under a 16-hour light/8-hour dark cycle with a relative humidity of 60%. The roots at the three- to four-leaf stage were collected for RNA extraction. Samples were obtained from three individual plants. A total of 3 μg RNA per sample was used for the RNA sample preparation. Sequencing libraries were generated using the NEBNext Ultra™ RNA Library Prep Kit for Illumina (NEB, USA) following the manufacturer’s recommendations, and index codes were added to attribute sequences to each sample. The fragments per million mapped reads per kilobase (FPKM) for each gene was calculated using HTSeq v0.6.1 (Trapnell et al., 2012), which was used to map to upland cotton transcripts with the aid of the *G. hirsutum* genome annotation.

Differential expression analysis of the two materials was performed using the DESeq R package (1.18.0) (Wang and Cairns, 2014). Genes with an adjusted *P*-value of <0.05 found by DESeq were assigned differentially expressed genes (DEGs). Gene Ontology (GO) enrichment analysis of DEGs was implemented in the GO-Seq R package (Wang and Cairns, 2014). GO terms with a corrected *P*-value less than 0.05 were considered significantly enriched by DEGs. KOBAS software (Mao et al., 2005) was used to test the statistical enrichment of DEGs in KEGG pathways (http://www.genome.jp/kegg/). The RNA-seq data were provided by the BioMarker (BMK) company (http://www.biomarker.com.cn/).

The data for eight high gossypol materials (SRS1691460, SRS1660920, SRS1660924, SRS1661035, SRS1691433, SRS1691495, SRS1660917, and SRS1691615) and two low gossypol materials (SRR10306147 and SRS1691645) were downloaded from NCBI. The FPKM values were analyzed by hisat2 (v2.2.1), samtools (v1.16.1), and stringtie (v2.2.1) to obtain the absolute expression levels of different expression genes.

RNA-seq data, including gene accession numbers, were available in the NCBI SRA under accession number PRJNA936998.

### RNA isolation and quantitative real-time PCR analysis

Protocols to isolate RNA and quantitative real-time PCR were followed as described previously (Zhao et al., 2016). We analyzed the dissociation curves for each reaction and used the 2^−ΔΔCT^ method to calculate the expression level of each target gene (Zhao et al., 2016). All reactions were conducted with at least three biological replicates. The relevant gene-specific primers in the experiments are listed in **Table S1**.

### Statistical analysis

Student’s t-test and Tukey’s ANOVA test were used to perform all statistical analyses and generate a *P*-value. The data represent the mean ± SD of n ≥3 independent experiments. All tests were two-tailed. The data were normalized, and all samples were normally distributed with homogeneity of variance.

## Results

### Phylogenetic analysis of *TPS* family genes

**Figure 1:**
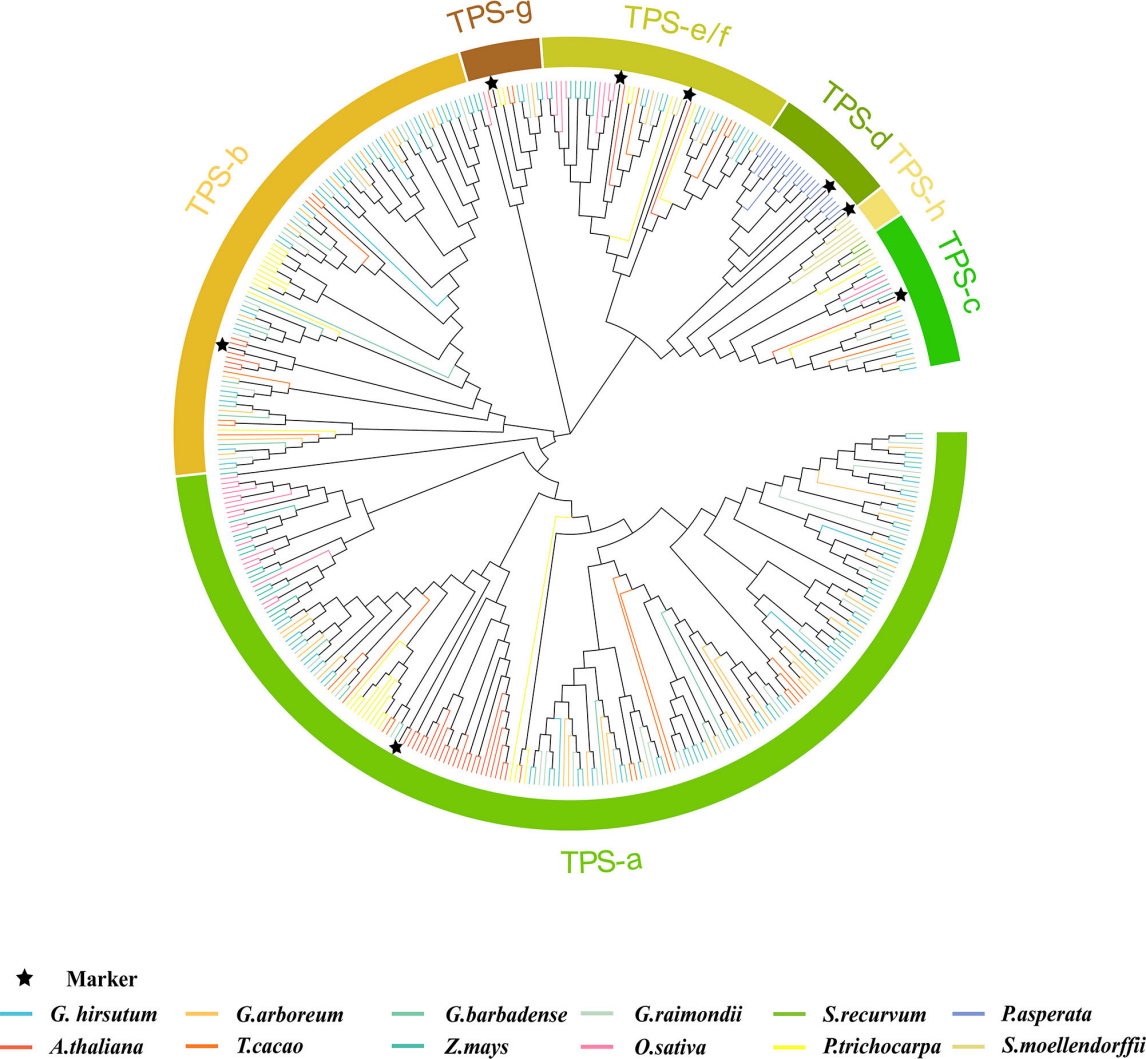
Phylogenetic analysis of the *TPS* gene family. MEGA7.0 was used to analyse the phylogenetic analysis of 430 sequences of 12 species: *Gossypium hirsutum*, *G. arboreum*, *G. barbadense*, *G. raimondii*, *Zea mays*, *Picea asperata*, *Arabidopsis thaliana*, *Theobroma cacao*, *Oryza sativa*, *Populus trichocarpa*, *Sphagnum recurvum*, and *Selaginella moellendorffii*. The *TPS* gene/protein in the published literature was used as control and labeled ★. The colors of different branches of the phylogenetic tree represented different species, the same color represented the same species, and different colors in the outer circle represented different subfamily classifications, which were divided into seven categories, namely *TPS-a*, *TPS-b*, *TPS-c*, *TPS-d*, *TPS-e/f*, *TPS-g*, and *TPS-h*.

**Figure 2:**
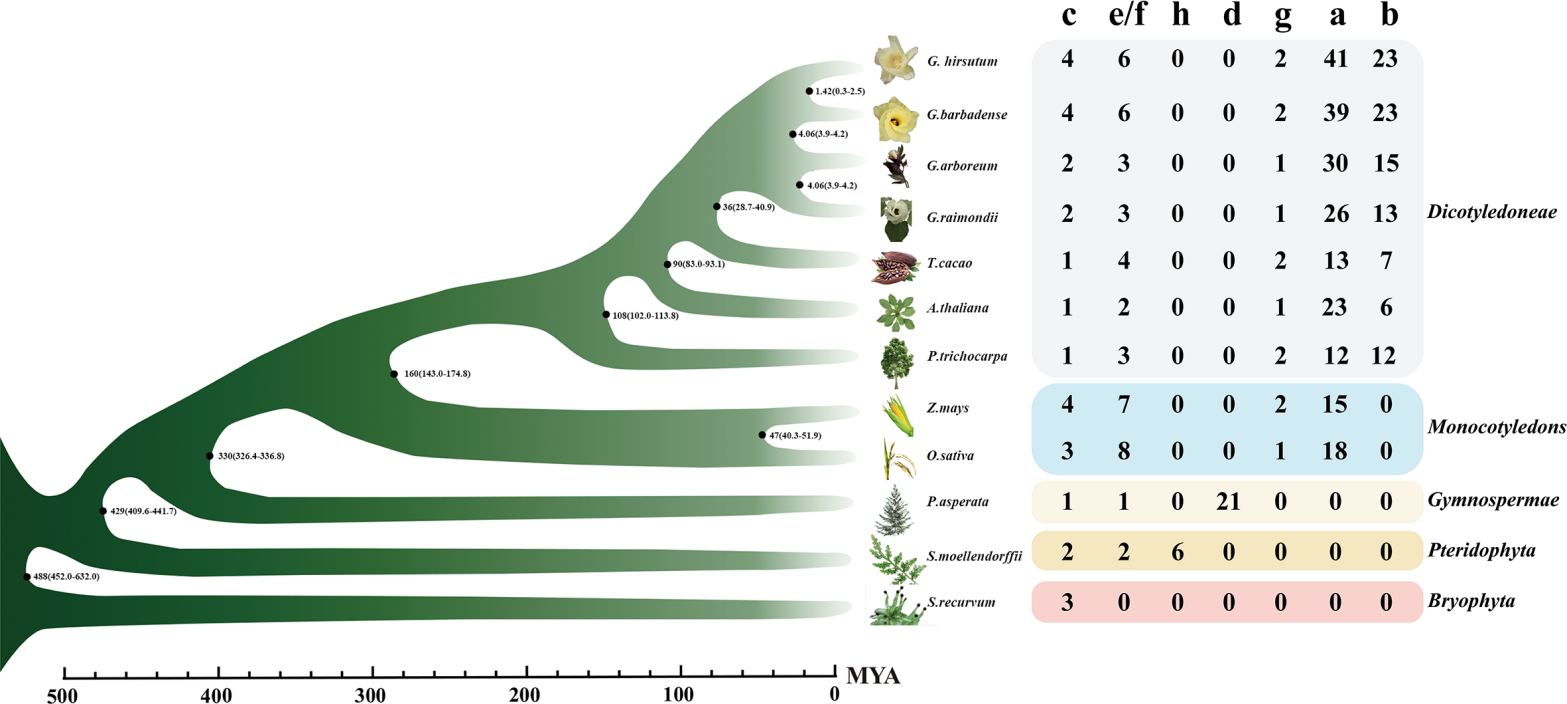
The numbers of the *TPS* gene family of 12 species. The bar value below indicated the time of species divergence. On the right side were species name, species, number of genes in different *TPS* subfamilies and species classification. Different colored squares indicated different species classifications. The phylogenetic drawing was based on timetree. MYA, A million years ago.

To find out the evolution mode of the *TPS* gene and various evolutions during the evolution process, the whole genome data of 12 land plant species were screened to analyze the evolution of the *TPS* family. With PF01397 and PF03936 as indexes according to the PFAM database, 528 sequences were screened out initially (**Table S2**), 430 of which were longer than the 500 amino acids (aa) that had been reported that can encode complete proteins (Chen et al., 2011). Based on previously reported *TPS* genes/proteins as markers (Aubourg et al., 2002), 430 *TPS* genes could be divided into eight subfamilies, namely *TPS-a* to *TPS-h*. However, other pieces of literatures combined *TPS-e* and *TPS-f* into a subgroup (Martin et al., 2004). Thereby, we also divided the 430 sequences into seven subfamilies (**Figure 1**), showing 217, 99, 28, 21, 45, 14, and 6 members in *TPS-a*, -b, -c, -d, -e/f, -g, and -h, respectively (**Figure 2**). Bryophytes, *S. recurvum*, had only three *TPS-c* genes, indicating that the *TPS-c* subfamily may be the earliest subfamily in land plant species, which was kept in 12 land plant species possibly for performing basic functions. Two *TPS-e/f* and six *TPS-h* are present in ferns, *S. moellendorffii*, of which the *TPS-e/f* subfamily is shared by gymnosperms and angiosperms while the *TPS-h* subfamily is absent in higher land plant species. Interestingly, 21 *TPS-d* were present in gymnosperms (*P. asperata*) but were lost in angiosperms, indicating that lots of *TPS-d* genes were required for gymnosperm plant growth and development. The loss of *TPS-d* and *TPS-h* subgroups in angiosperms reflected the historical records that gymnosperms had undergone many major changes in the geology and climate since their emergence. In angiosperms, the *TPS-a*, *TPS-b*, and *TPS-g* subfamilies were derived. The *TPS-b* subfamily genes were reported to distribute in dicotyledons, excluding an example of *TPS-b* presence in the monocotyledonous plant sorghum (Chen et al., 2011). Overall, seven subfamilies of *TPSs* had diverged over a long evolutionary history with the lineage-specific expansion of certain subfamilies. *TPS-a* had the largest number of *TPS* genes in monocotyledonous and dicotyledonous plants, so it was worthy of exploration.

### Collinearity analysis of *TPS* family genes in *G. hirsutum*, *G. raimondii*, and *G. arboreum*

In three cotton species, the *TPS* family genes contained five subfamilies: *TPS-a*, *TPS-b*, *TPS-c*, *TPS-e/f*, and *TPS-g*, of which the *TPS-a* and *TPS-b* subgroups contained the most members (**Figure 2**). The tetraploid *G. hirsutum* was reported to derive from the doubling of diploid *G. raimondii* and *G. arboreum* 1.5 million years ago (Hu et al., 2019). Therefore, the number of these five *TPS* subfamilies in *G. hirsutum* should theoretically be the sum of the *G. raimondii* and *G. arboreum* members. Conversely, only the *TPS-c*, *TPS-e/f*, and *TPS-g* subfamilies conformed to this rule, indicating that the three subfamilies were highly conserved among the three cotton species. The *TPS-a* and *TPS-b* subfamilies did not conform to this rule, which preliminarily suggested that large numbers of duplications and loss cases might have occurred during their evolution.

**Figure 3:**
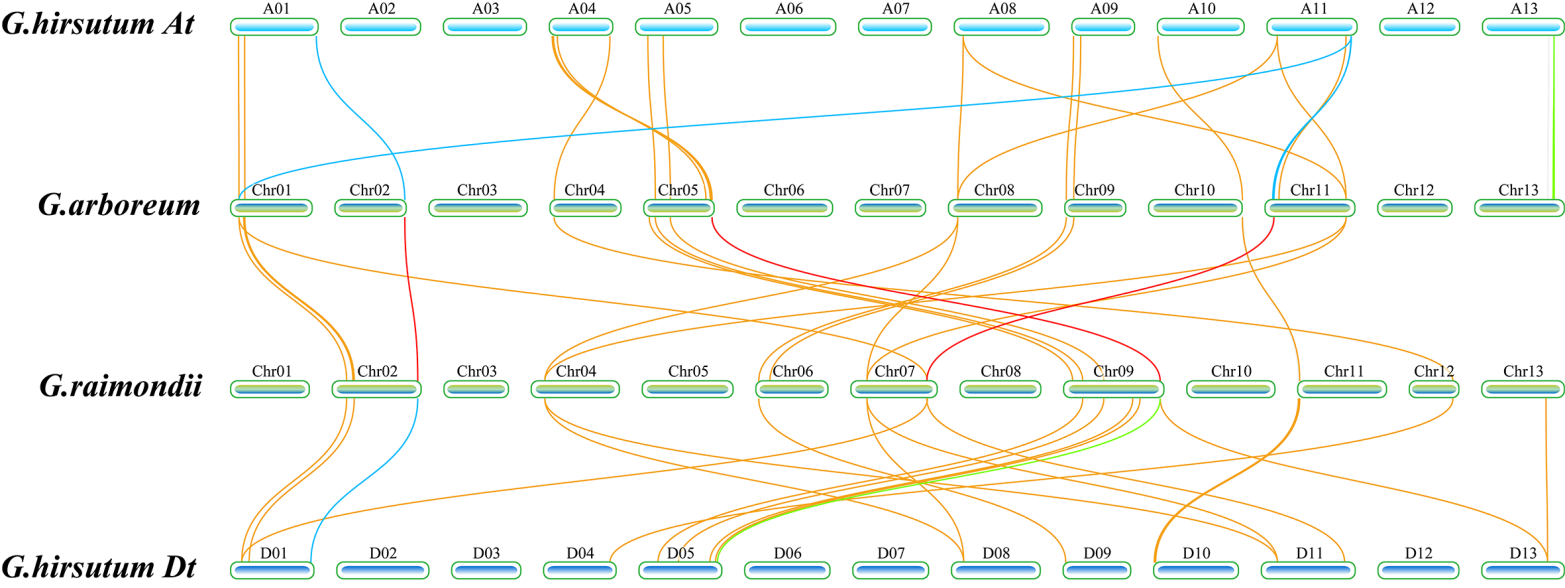
Schematic diagram of collinear analysis of the *TPS* family genes of *G. hirsutum* A/D subgroup and *G. arboreum* and *G. raimondii*, respectively. The blue squares represented the chromosome of *G. hirsutum*, the At subgroup of *G. hirsutum* was at the top, and the Dt subgroup of *G. hirsutum* was at the bottom. In the figure, the green squares represented the chromosomes of *G. arboreum* and *G. raimondii*, and the length represents the length of the chromosome. The name of the chromosome was labeled above each chromosome. The lines in the figure represented collinear relationships between species. The yellow lines represented collinear relationships between *G. hirsutum* and both *G. arboreum* and *G. raimondii*, the red lines represented collinear relationships of *GhCDNs* between *G. arboreum* and *G. raimondii*, the blue lines represented collinear relationships of 12 *GhCDN-A* between *G. hirsutum* and both *G.arboreum* and *G. raimondii*, the green lines represented collinear relationships of other *GhCDNs* between *G. hirsutum* and both *G.arboreum* and *G. raimondii*, and the positions of the lines on chromosomes represented the relative positions of genes on chromosomes.

To reveal whether replication and loss have occurred, we performed a colinear analysis of all *TPS* genes in diploid *G. arboreum* and *G. raimondii* and tetraploid *G. hirsutum* (**Figure 3**). When PF01397 and PF03936 were used as indexes to scan the whole genome for identification of *TPS* family genes, it was found that there were 75 *TPS* genes in *G. arboreum*, 60 in *G. raimondii*, and only 91 in *G. hirsutum* without any filtration (Huang et al., 2018; Cui et al., 2023). Collinearity analysis showed that 22 sequences showed collinearity between *G. arboreum* and *G. hirsutum*, and 20 sequences showed collinearity between *G. raimondii* and *G. hirsutum*. According to the longer than 500 aa standard, 51, 45, and 76 *TPS* genes were screened out in *G. arboreum*, *G. raimondii*, and *G. hirsutum*, respectively, indicating that other genes’ functions might have been lost in the process of evolution to adapt to environmental changes. Therefore, 22 longer *TPS* genes were collinear within *G. arboreum* and *G. hirsutum*, and 20 *TPS* genes were collinear within *G. raimondii* and *G. hirsutum*, indicating that the contributions of *G. arboreum* and *G. raimondii* seemed to be similar in the *TPS* genes of *G. hirsutum*. The data also indicated that most of the *TPS* family genes with collinearity were above 500 aa. Many *TPS* genes, including subfamilies *TPS-a* and *TPS-b*, showed no collinearity among the three cotton species (**Table S3**), possibly resulting from adaptation to the different environmental pressures.

Among the 22 collinear genes between *G. hirsutum* and *G. arboreum*, 10 *TPSs* belong to the *TPS-a* subfamily, and six *TPSs* belong to the *TPS-b* subfamily. Among the 20 collinear genes between *G. hirsutum* and *G. raimondii*, eight *TPSs* belong to the *TPS-a* subfamily, and six *TPSs* belong to the *TPS-b* subfamily. However, there were only 38 *TPS* genes in *G. hirsutum* that were collinear with *G. arboreum* or *G. raimondii*, including 15 *TPS-a* genes and 13 *TPS-b* genes (**Table S3**). The results suggested that a large number of duplications and losses occurred in the *TPS-a* subfamily, whereas the *TPS-b* subfamily genes had better integrity in the evolution process of *G. hirsutum*.

Tandem and segmental duplications significantly promoted the expansion and the expression differentiation of *TPS* gene families in *G. hirsutum*, as shown in **Figure S1**. Then we employed TBtools to calculate d_S_ (synonymous substitutions) and d_N_ (non-synonymous substitutions) values (**Table S4**). The d_N_/d_S_ ratio of all duplicate gene pairs was less than 1, indicating that the duplicate gene pairs had undergone a purification and selection process (Goldman and Yang, 1994).

### Evolution and structure analysis of *GhCDNs* (cadinene synthase) subfamily genes

Given that many duplications, losses, and selections occurred in the *GhTPS*-a subfamily during evolution, further research was mainly focused on the *GhTPS*-a subfamily genes. Previous reports showed that *TPS-a* could be divided into two branches, a1 and a2, and the a1 branch was composed of dicotyledonous plants, while a2 was composed of monocotyledonous plants (Chen et al., 2011). Therefore, *TPS*-a genes of dicotyledonous plants, including cotton and monocotyledonous plants were constructed into the phylogenetic tree. The results confirmed this notion that *GhTPS-a* genes belong to the a1 branch (**Figure S2**).

**Figure 4:**
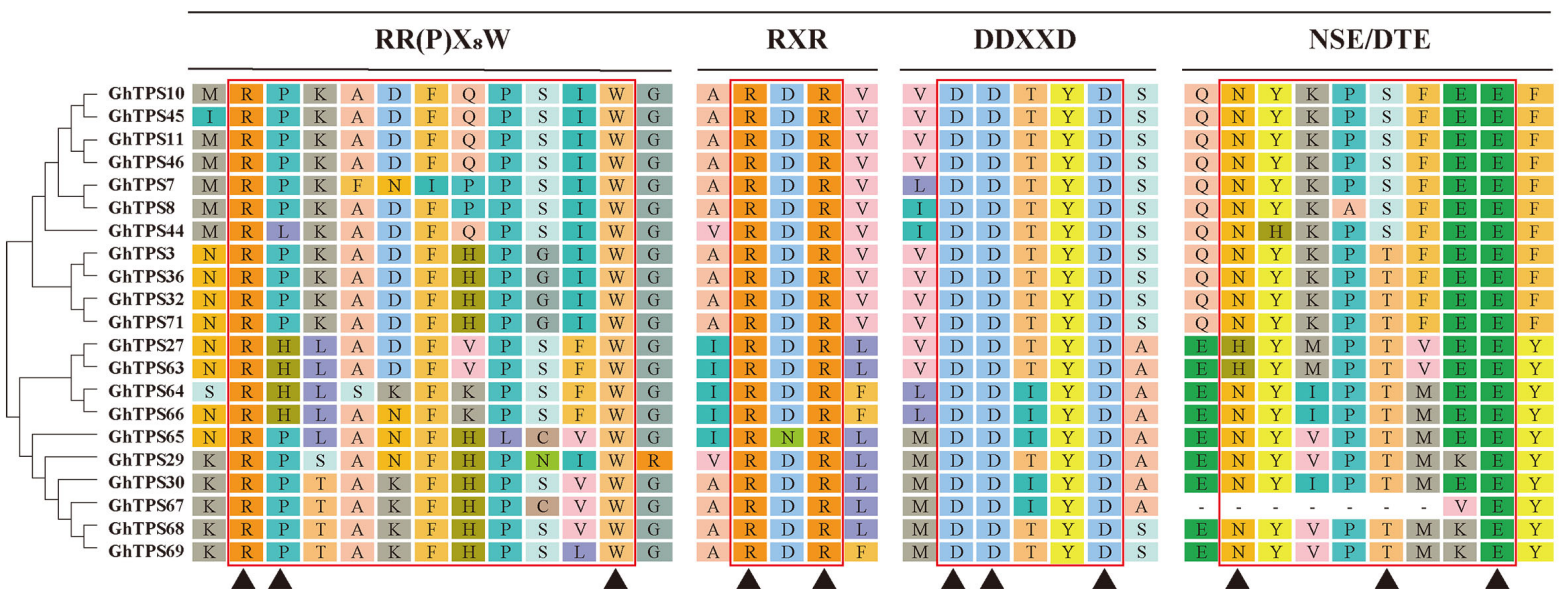
Schematic diagram of *GhTPS-a* conservative motif in *G. hirsutum.* On the left was *GhTPS-a* phylogenetic tree analysis of *G. hirsutum*. The red line was the conserved motif, the name of the conserved motif was indicated above, and ▲ was the conserved amino acid site.

Based on analysis of the conserved motif, the *GhTPS*-*a1* gene subfamily contains a conservative RR(P)X_8_W motif (PF01397) at the N-terminus and a conservative DDXXD and NSE/DTE motif (PF03936) at the C-terminus, similar to the previous report (Degenhardt et al., 2009). The RR(P)X_8_W motif (PF01397) mainly had a role in the cyclization of terpenoids, and loss of this motif results in acyclic volatile terpenoids. The DDXXD motif was a metal-binding motif that could bind metals, which is enhanced by the NSE/DTE structure with metal ligands. In addition, a RXR motif was found in the *GhTPS*-a1 subfamily (**Figure 4**) and was reported to be related to the di-salt complexation reaction after substrate ionization (Starks et al., 1997).

By functional annotation of *GhTPS-a1* subfamily genes, 21 of the 41 *GhTPS-a1* genes belong to the *GhCDN* (*cadinene synthase*) gene subfamily supported by the phylogenetic tree (**Figure S2**, **Table S5**). According to evolutionary analysis, these *CDN* family genes were divided into five subfamilies, A, B, C, D, and E, among which the *CDN-B* and *CDN-D* subfamilies were reported to be almost pseudogenes (Sun et al., 2010; Cheng et al., 2016), and the *CDN-C* subfamily had the most members (Townsend et al., 2005). There are 12, 1, 5, 1, and 2 members in the *GhCDN*-A, B, C, D, and E subfamilies, respectively. The collinearity analysis showed that six of the 21 *GhCDNs* had collinearity with *G. arboreum* or *G. raimondii*, of which four *GhCDN-A* genes were collinear and all had tandem duplications or segmental duplications. Therefore, we will further focus on research on the *GhCDN-A* subgroup genes.

### Bioinformatic analysis and expression pattern analysis of *GhCDN-A* family genes

To evaluate the *GhCDN-A* characterization in cotton, we performed an analysis of chromosomal location. Twelve *GhCDN-A* genes were mainly distributed on chromosome 1 (two genes) and chromosome 11 (10 genes) (**Table 1**), which is different from the chromosome distribution of the 76 *TPS* genes (Karaca and Ince, 2023) (**Figure S3**; **Table S6**). The length of GhCDN-A amino acids ranged from 505 to 1,027, the molecular weight ranged from 58.51 kDa to 117.35 kDa, the isoelectric points ranged from 4.91 to 6.65, and the hydrophilicity ranged from −0.481 to −0.163. The instability index of GhCDN-A proteins was high, and they could be mainly located in the cytoplasm. Structural analysis revealed that the *GhCDN-A* genes had six introns and seven exons (**Figure S4**), whose encoding proteins contained four conserved motifs: RR (P)X_8_W, RXR, DDXXD, and NSE/DTE (**Figure 4**).

**Table 1:** Bioinformatics analysis of the *GhCDNA* gene family in *G. hirsutum*.

		Gene Id	Number of amino acids	Molecular weight	Isoelectric point	Instability index	Average hydrophilicity	Subcellular localization	Chromosome position	Gene location		Gene strand	CDS length
GhCDNA1	GhTPS3	Gh_A01G225600	556	64.54	5.06	40.61	-0.40	Chloroplast.	A01	111872920	111875125	–	1668
GhCDNA2	GhTPS27	Gh_A11G315500	519	60.63	5.50	40.79	-0.28	Chloroplast.	A11	109757997	109761972	+	1557
GhCDNA3	GhTPS29	Gh_A11G316100	532	61.92	5.87	42.23	-0.32	Chloroplast.	A11	109996811	109999618	+	1596
GhCDNA4	GhTPS30	Gh_A11G316500	558	64.53	5.39	40.82	-0.28	Chloroplast.	A11	110077361	110080888	+	1674
GhCDNA5	GhTPS36	Gh_D01G221400	556	64.47	4.94	43.24	-0.38	Chloroplast.	D01	61187935	61190135	–	1668
GhCDNA6	GhTPS63	Gh_D11G316000	556	65.04	5.39	38.88	-0.27	Chloroplast.	D11	63451758	63455577	+	1668
GhCDNA7	GhTPS64	Gh_D11G316300	559	64.86	5.64	42.97	-0.34	Chloroplast.	D11	63512829	63516510	+	1677
GhCDNA8	GhTPS65	Gh_D11G316700	565	65.60	5.67	42.17	-0.34	Chloroplast.	D11	63602391	63606311	–	1695
GhCDNA9	GhTPS66	Gh_D11G316800	554	64.10	5.44	46.62	-0.31	Chloroplast.	D11	63615241	63620250	+	1662
GhCDNA10	GhTPS67	Gh_D11G317500	505	58.51	6.43	39.98	-0.35	Chloroplast.	D11	63755439	63758723	+	1515
GhCDNA11	GhTPS68	Gh_D11G317700	556	64.16	5.31	44.24	-0.28	Chloroplast.	D11	63800065	63803384	+	1668
GhCDNA12	GhTPS69	Gh_D11G317900	559	64.88	5.53	43.98	-0.32	Chloroplast.	D11	63881918	63884870	+	1677

**Figure 5:**
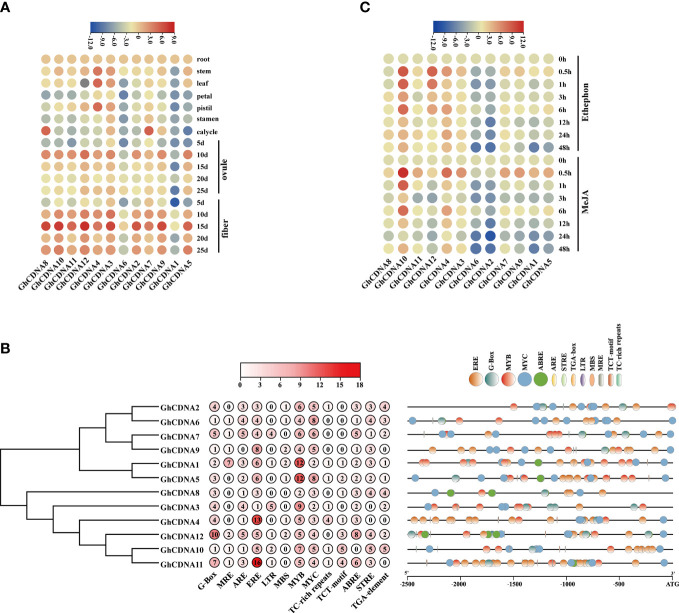
The promoter analysis and expression analysis of *GhCDN-A* gene family. **(A)** Heatmap of the quantitative real-time PCR of 12 *GhCDN-A* genes in 17 tissues. **(B)** The analysis of 2.5 kb promoter of 12 *GhCDN-A* genes, which successively shows the phylogenetic tree of *GhCDN-A* genes, the number of *cis*-regulatory elements, and the number of *cis*-regulatory elements. **(C)** Heatmap of the expression level of *GhCDN-A* genes in roots after treating with 50 μM MeJA and 50 μM ethephon.

According to the qRT-PCR analysis, *GhCDN-A* genes had high expression levels in seeds, roots, hypocotyls, and stems (**Figure 5A**), a consistent trend with the published transcriptome data with slight differences in various tissues and organs (**Figure S5**) (Zhang et al., 2015).

To investigate the regulation of expression of *GhCDN-A* genes, the 2.5kb promoter region was identified by *cis*-regulatory element analysis. By screening the *cis*-regulatory elements appearing in more than four genes, the promoter region of *GhCDN-A* genes contained light cycle-related elements, defense and stress-related elements, hormonal responses-related elements, growth and development-related elements, etc. (**Figure 5B**). The defense and stress-related elements, including G-box and MYC elements, play an important role in the JA signaling pathway. ERE, as an ethylene-induced *cis*-regulator*y* element and MYB transcription factor binding elements were present in the promoters of all *GhCDN-A* genes. The special *cis*-elements in the promoters of *GhCDN-A* genes suggested that the JA and ethylene signaling pathways may be involved in gossypol synthesis as well as the MYB transcription factor. According to the map of the location and quantity of *cis-*regulatory elements, the promoters of 12 *GhCDN-A* genes contain different *cis*-elements, indicating that *GhCDN-A* genes have different expression profiles.

To verify whether *GhCDN-A* genes were induced by MeJA and ethephon, their expression levels were tested under the application of exogenous hormones. The results showed that three *GhCDN*-A genes, *GhCDNA4*, *GhCDNA10*, and *GhCDNA12*, were upregulated by ethephon, whereas *GhCDNA1*, *GhCDNA2*, *GhCDNA5*, *GhCDNA6*, and *GhCDNA9* were downregulated. Under MeJA treatment, *GhCDNA4* and *GhCDNA10* were upregulated, while *GhCDNA1*, *GhCDNA2*, *GhCDNA5*, *GhCDNA6*, and *GhCDNA9* were downregulated (**Figure 5C**). This result indicated that several key *GhCDN-A* genes were induced by ethephon and MeJA to possibly participate in plant defense.

### The *GhCDN-A* subfamily genes participate in gossypol synthesis

To investigate the relationship between the *GhCDN-A* subfamily genes and gossypol content, the expression levels of the *GhCDN-A* subfamily genes were analyzed in eight high-gossypol and two low-gossypol varieties based on the published article (Wang et al., 2017). Interestingly, there were comparable expression levels in *GhCDN-A* subfamily genes among high/low gossypol varieties (**Figure S6A**), indicating that they participate in gossypol synthesis and are not due to a single major gene.

**Figure 6:**
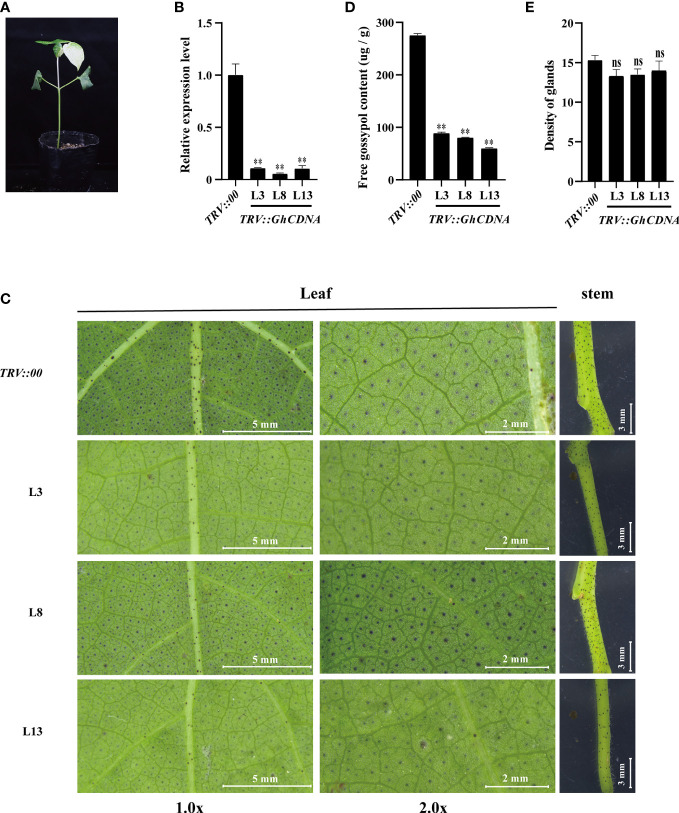
Knockdown of the *GhCDNA* subfamily genes reduced the gossypol content. **(A)** The phenotypes of *TRV::PDS.* **(B)** The silencing efficiency of the *GhCDNA* subfamily genes in *TRV::00* and *TRV::GhCDNA* plants. *L3*, *L8*, and *L13* represented three different strains of *TRV::GhCDNA* plants. **(C)** The phenotypes of *TRV::00* and *TRV::GhCDNA* plants. From left to right, the main vein area of cotton leaves was shown, the cotton leaves magnified twice was shown, and the upper node area of cotton stem was shown, with the relative bar value in the lower right corner. **(D)** The gossypol content in *TRV::00* and *TRV::GhCDNA* plants. **(E)** The density of glands *TRV::00* and *TRV::GhCDNA* plants. Bars represent SDs (standard deviation) of three independent biological replicates. The single asterisk indicates statistical significance at P <0.05. The double asterisk indicates statistical significance at P <0.01.

To further evaluate the *GhCDN-A* function in gossypol synthesis, we employed two key genes, *GhCDN-A1* and *GhCDN-A5*, to separately knock them down *via* the tobacco rattle virus (TRV)-induced gene silencing (VIGS) method. Gene-specific fragments were inserted into the p156 vector to construct *TRV: GhCDN-A* silencing vectors. The 7-day-old cotton seedlings were agroinfiltrated with a needleless syringe and a corresponding gene silencing vector with the help of the p192 vector. The *GhPDS*-silencing plants were used as positive controls, while plants infected with empty vectors were regarded as negative controls. After 14 d post infection (dpi), the *GhPDS*-silencing plants (a positive control) showed a bleaching phenotype (**Figure 6A**), while silencing efficiency in *GhCDN-A* gene-silencing plants was tested by qPCR analysis. The corresponding target expression levels of *GhCDN-A* gene-silencing plants significantly decreased compared to the control treated with an empty vector (**Figure S6B**). Compared to the control, the density of glands and the gossypol contents in two *GhCDN-A* gene-silencing leaves did not significantly change (**Figures S6C, D**). Then, we divided all 12 *GhCDN-A* genes into three groups to develop multi-gene-silencing plants; there was also no significant change in the density of glands or the gossypol content between these gene-silencing leaves and the control (**Figures S6E–G**). Finally, we developed all 12 *GhCDN-A*-silencing plants (**Figure S7**). The plants with total silencing efficiency below 20% were screened to evaluate glands and gossypol accumulation (**Figure 6B**). As shown in **Figure 6**, there was no significant change in the density of glands in three 12 *GhCDN-A* gene-silencing leaves L3, L8, and L13, compared to the control, but the glands became lighter in color. In line with the results, the gossypol content in the *TRV::GhCDN-A* plants decreased by 70% compared to the control, according to HPLC analysis. The results showed that *GhCDN-A* participated in the gossypol synthesis with functional redundancy or quantitative effect.

### Revealing key genes of gossypol synthesis based on RNA-seq

To further elucidate the mechanism underlying regulation of *GhCDN-A* subfamily genes in gossypol synthesis, two extreme varieties with and without gossypol in glands (thereafter glandular (G) and glandless (GL)) were used for transcriptome analysis at three repeats. The correlations of the transcript data from six samples were all above 0.99, indicating good repeatability and high reliability (**Figure S8A**). Under the conditions of FDR ≤0.01 and log_2_|FC|≥1, 1,193 differentially expressed genes (DEGs) were screened (**Figure S8B**). Enriching these DEGs, we found that most of the DEGs were enriched in plant–pathogen interaction, plant defense, and secondary metabolite synthesis (**Figures S8C, D**).

**Figure 7:**
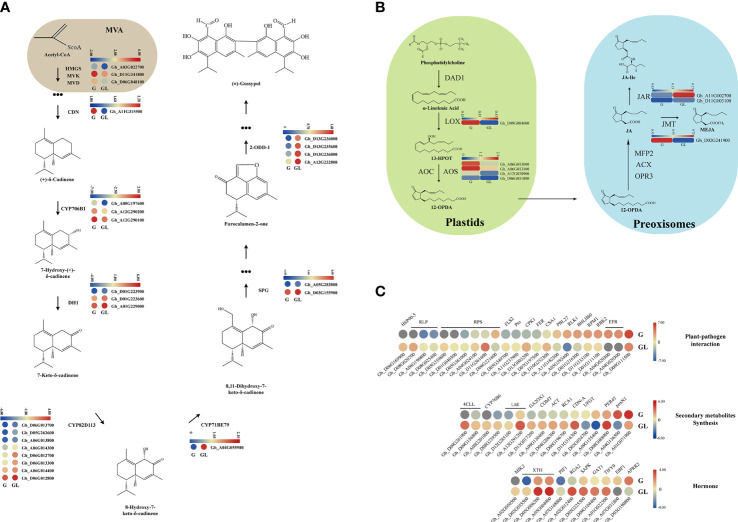
Heatmap of the differentially expressed genes assigned to gossypol biosynthetic and jasmonic acid biosynthetic pathways. **(A)** The gray part was MVA pathway, the other part was the gossypol biosynthetic pathway. **(B)** The jasmonic acid biosynthetic pathway, which were in the plastid and deoxyridisome, respectively. **(C)** The differentially expressed genes were involved in plant pathogen interaction, secondary metabolic synthesis and hormone synthesis. Only the differentially expressed genes in gossypol and non-gossypol materials were shown in the figure. The genes in the red box were upregulated without treatment, and the blue solid line were downregulated. G, glandular cotton; GL, glandless cotton; HMGS, hydroxymethylglutaryl-CoA synthase; MVD, mevalonate 5-diphosphate decarboxylase; MVK, mevalonate kinase; CDN, (+)-δ-cadinene synthase; DH, short-chain alcohol dehydrogenase; SPG, specialized glyoxalase; 2-ODD, 2-oxoglutarate/Fe (II)-dependent dioxygenase.

As shown in **Figure 7A** on the gossypol synthesis total process, one *HMGS*-coding gene (Gh_A03G022700), one *MVK* gene (Gh_D11G341800), and one *MVD* gene (Gh_D06G048100) at the beginning of the gossypol synthesis pathway (MVA pathway) were significantly upregulated in expression levels in the glandular cotton variety compared to the glandless variety. Then, a key gene, *GhCDN2* (Gh_A11G315500), and three CYP706B1-coding genes (Gh_A08G197600, Gh_A12G290200, and Gh_A12G290100) in the gossypol synthesis pathway were also upregulated in the glandular variety. Conversely, from 7-Hydroxy-(+)-δ-cadinene to deoxy-hemi-gossypol, three DH1 coding genes (Gh_D01G223900, Gh_D01G223600, and Gh_A01G229000), eight CYP82D113 genes (Gh_D06G013700, Gh_D05G262600, Gh_A06G013800, Gh_A06G014300, Gh_D06G012700, Gh_D06G013300, Gh_A06G014400, and Gh_D06G012800), one CYP71BE79 gene (Gh_A04G055900), two SPG genes (Gh_A05G283000 and Gh_D03G155900), four 2-ODD-1 coding genes (Gh_D13G236000, Gh_D13G235600, Gh_D13G236000, and Gh_A13G232800) were upregulated to different degrees in glandless cotton variety. It is interesting why these late gossypol synthesis genes are upregulated in the glandless variety compared to the glandular variety, which is worthy of further exploration in the future.

Some pieces of literatures have demonstrated that *Gossypium PIGMENT GLAND FORMATION GENE* (*GoPGF*) (as a regulator) feedbacks on the gossypol biosynthesis pathway through regulating the expression of JAZ, WRKYs, or other genes, which are induced by exogenous signals including JA (Chen et al., 1995; Xiao-Ya et al., 1996). Based on the RNA-Seq data, the LOX encoding genes (Gh_D09G084000) in the JA synthesis pathway and the AOS encoding genes (Gh_A06G013000, Gh_A06G013100, Gh_A12G030900, and Gh_D06G011800) were significantly downregulated in the glandless variety, indicating that JA had a positive effect on the gossypol synthesis pathway (**Figure 7B**).

It had been reported that gossypol was mainly involved in plant resistance and insect resistance (Puckhaber et al., 2002; Townsend et al., 2005; Stipanovic et al., 2006; Turco et al., 2007; Mellon et al., 2012; Gao et al., 2013). The results of enrichment analysis also indicated that gossypol appears to play an important role in plant resistance. Many star genes in the plant defense response, including *FLS2*, *RLP*, *RPS*, *CPK1*, *RBK2*, *EFR*, *BHLH60*, etc., showed differential expression between glandular and glandless varieties. Most *RLP*, *RPS*, and *FLS2-*encoding genes showed downregulated expression in the glandular variety, whereas other genes showed upregulated expression (**Figure 7C**). Therefore, these data confirm that gossypol participates in plant defense.

In the glandless variety, other secondary metabolism synthesis-related genes, including *COMT*, *ACT*, *PRE45*, etc., showed an upward trend compared to the glandular variety, indicating that other secondary metabolites synthesis may be promoted in glandless cotton. In this data, we found that most phytohormone signaling-related genes, including the JA pathway (*TIFY9*) and ethylene (*EBF1*), showed an upward trend in glandless cotton (**Figure 7C**). Our previous transcriptome data on the comparison of *JAZ1/TIFY10A*-overexpression plants and wild-type plants showed that several *GhCDN-A* genes were upregulated (**Figure S9**), further indicating that JA may participate in the synthesis of gossypol, which is consistent with the previous report (Li et al., 2014).

## Discussion

### The evolutionary of *TPS* family genes

As one of the most diverse compounds in plant secondary metabolism, terpenoids have been widely used in spices, medicine, disease resistance, and other fields (Yamada et al., 2012; Schmidt-Dannert, 2015; Tholl, 2015; Beran et al., 2016; Christianson, 2017). The *TPS* family is highly conserved in structures, motifs, and functions in reported plants, including almond (Nawade et al., 2019), tomato (Falara et al., 2011), poplar (Irmisch et al., 2014), *Arabidopsis* (Aubourg et al., 2002), grape (Martin et al., 2010), and rice (Chen et al., 2014), while it is not in algae (Hernandez-Garcia et al., 2021). There is only *TPS-c* in bryophytes, indicating that this subgroup is one of the earliest. The derivation of bryophytes reveals the gradual transition from aquatic plants to terrestrial plants, during which *TPS-c* is conserved, indicating that it may be involved in the synthesis of ancient gibberellin (Hernandez-Garcia et al., 2021). Besides the *TPS-c* subgroup, the *TPS-h* subgroup was also derived from *S. moellendorffii* and was suggested to be involved in its emergence (Chen et al., 2011). The terpenoid synthase derives the d and e/f subfamilies in gymnosperms, while the *TPS-h* subfamily in pteridophytes was lost, which may be related to the appearance and evolution of gymnosperms after several major changes in the geological climate since gymnosperms came into being (Wan et al., 2022). In angiosperms, the *TPS-d* subfamily derived from gymnosperms disappeared, leaving the unique *TPS-a*, *TPS-b*, and *TPS-g* subfamilies. At present, no species that can contain seven *TPS* subfamilies have been found, indicating that there has been differentiation of *TPS* subfamilies in a long evolutionary history. Some species exhibit lineage-specific dilatation in certain subfamilies, with large family dilatation occurring mainly in dicotyledon and monocotyledon plants (**Figures 1**, **2**).

### The identification and analysis of *GhCDN* family genes

According to the latest genomic data (Yang et al., 2019), we found that *G. hirsutum* contains 76 full-length *GhTPSs*, which can be divided into five subfamilies: *TPS-a*, *TPS-b*, *TPS-c*, *TPS-e/f*, and *TPS-g* (**Figure 2**). Among them, *TPS-c*, *TPS-e/f*, and *TPS-g* subfamily genes in cotton could be collinear with *G. arboreum* and *G. raimondii* (**Figure 3**). However, the *TPS-a* and *TPS-b* subfamilies have been replicated and lost in large quantities, which is mainly due to the expansion of genes by tandem and fragment replication and the screening of genes to adapt to changes in the environment by purification selection (**Figures S2**, **S3**). It also indicated the high conservation and diversity of *TPS-a/b* during the formation of *TPS* family members.

The *GhCDN* gene was a key enzyme involved in the gossypol synthesis pathway. As a branch of the *GhTPS-a1* subfamily, the *GhCDN* gene family also replicates through fragment duplications and tandem duplications. In the process of replication, some parts of the gene were lost, resulting in the generation of non-functional pseudogenes, such as the *GhCDN-B* subfamily genes. A relatively complete copy of most of the genes was produced to produce functional genes, such as *GhCDN*-*A*, *C*, *D*, and *E*. It was reported that there was one member of the *CDN*-*A* subfamily in *Arabidopsis* (Aubourg et al., 2002), but 12 members had been identified in *G. hirsutum*, indicating that a large number of gene replications occurred in this subfamily (**Figure 4**). Therefore, our research focus was mainly on *GhCDN*-*A*.

### *GhCDN-A* family genes involved in gossypol synthesis

*GhCDN-A* may be induced by MeJA and ethephon (**Figures 5B, C**), which is consistent with previous reports (Li et al., 2014). The VIGS experiment proved that all 12 *GhCDN-A* gene plants showed significantly lower content of gossypol compared to the control, but the density of glands did not change (**Figure 6**), which indicated that the *GhCDN-A* family genes possess functional redundancy or quantitative effects. This preliminary result showed that the synthesis of gossypol had no obvious influence on the formation of glands, indicating independence, while the loss of glands had a certain degree of influence on the synthesis of gossypol, indicating that there is a certain relationship between them (Ma et al., 2016).

The transcriptome analysis of two extreme materials (with/without gossypol) for transcriptomic analysis showed that the DEGs were mainly enriched in plant–pathogen interaction, plant defense, and secondary metabolite synthesis, which was consistent with previous reports that gossypol was mainly involved in plant disease resistance, insect resistance, and other stress defenses (Puckhaber et al., 2002; Townsend et al., 2005; Stipanovic et al., 2006; Turco et al., 2007; Mellon et al., 2012; Gao et al., 2013). Additionally, the analysis of promoter elements indicated that JA may be involved in the regulation of gossypol formation, and *TIFY9* in the JA signaling pathway was significantly different between gossypol and gossypol-free materials, and *GhCDN-A* was upregulated in *JAZ1* (*TIFY10A)* overexpression materials previously reported (**Figure S9**). Therefore, we speculate that JA participates in the synthesis of gossypol. The results provided a meaningful reference for gossypol research in *G. hirsutum* and showed that *GhCDN-A* subfamily genes play an important role in gossypol synthesis.

## Conclusion

TPS proteins in plants are mainly involved in secondary metabolites and hormone synthesis, while in cotton, TPS is also involved in the synthesis of gossypol. Based on an evolutionary perspective, from mosses to ferns, gymnosperms, and angiosperms, *TPS* genes not only display conservative types but also evolve into new subfamilies through gene replication and loss. The *TPS* family can be divided into seven subfamilies, and *TPS-a* has the most members. 21 out of the 41 *TPS-a* genes in *G. hirsutum* belong to the *GhCDN* (cadinene synthase) gene subfamily and were divided into five subfamilies, A, B, C, D, and E. The special *cis*-elements in the promoters of 12 *GhCDN-A* genes suggested that the JA and ethylene signaling pathways may be involved in their expression regulation. Only silencing all genes of the *GhCDN-A* subfamily, the gossypol content in the plant was significantly reduced. All in all, these results reveal plant *TPS* gene evolution rules and assure that the *TPS* subfamily, *GhCDNA*, functions in gossypol synthesis in cotton.

## Data availability statement

The data presented in the study are deposited in the NCBI repository, accession number PRJNA936998.

## Author contributions

TW, JW, and GZ conceived and designed the experiments. TW, XX, and AR performed the experiments. TW and GZ analyzed the data. TW, GZ, and JW wrote the paper. GZ and JW revised the paper. All authors contributed to the article and approved the submitted version.

## Supplementary material

Supplementary Figure 1 Diagram of *GhTPSs* gene replication. The figure showed the result of collinear analysis of genes in *G. hirsutum*. The squares on the ring edge represent chromosomes, the names of chromosomes were marked on the top of chromosomes, the length of small squares represents the relative length of chromosomes, and the length was marked on the chromosomes, and the grey lines represented collinear relationship between all the *GhTPSs*, the blue lines represented collinear relationships between *GhCDNs*.

Supplementary Figure 2 Phylogenetic analysis of *TPS-a* subfamily genes. The branches with purple and pink background were *TPS-a1*, namely dicotyledonous plants, The branch with green background was *TPS-a2*, the monocotyledons. The branch with purple background was *CDN* gene. Different coloured dots represent different species, and species names are indicated below.

Supplementary Figure 3 Chromosome distribution of *GhTPS* gene family members in *G.hirsutum.* The chromosomal position was marked above each chromosome, the length of the short rod represented the length of the chromosome, the relative position of the gene was marked on the right side of the chromosome, the red line represented collinear between the two genes.

Supplementary Figure 4 The gene structure of *GhCDN-A.* The left was the *GhCDN-A* gene phylogenetic tree, the right was the gene structure, the green was the exon, the line was the intron, the blue was the untranslated region (UTR) and the length was the relative length of the gene.

Supplementary Figure 5 Heatmap of the expression level of *GhCDNs* in different tissue. The relative expression of *GhCDNs* in allotetraploid cotton (*Gossypium hirsutum L*. acc. TM-1) based on a previous report.

Supplementary Figure 6 Knockdown several key genes/cluster of *GhCDN-A* subfamily. Heat map of FPKM of 12 *GhCDNA* genes in eight high gossypol and two low gossypol varieties. **(A)** The silencing efficiency of *GhCDNA1* or *GhCDNA5* genes silencing plants. **(B)** The density of glands of leaves on *GhCDNA1* or *GhCDNA5* genes silencing plants. **(C)** The phenotypes of *GhCDNA1* or *GhCDNA5* genes silencing plants. **(D)** The silencing efficiency of three *GhCDN-A* gene clusters. The group I gene cluster contained *GhCDNA3, GhCDNA4, GhCDNA11, GhCDNA10, GhCDNA8, GhCDNA12*. The group II contained *GhCDNA1, GhCDNA5.* The group III contained *GhCDNA2, GhCDNA6, GhCDNA7*, and *GhCDNA9.* **(E)** The density of glands of leaves on three *GhCDN-A* gene clusters silencing plants. **(F)** The phenotypes of leaves on three *GhCDN-A* gene clusters silencing plants. Bars represent SDs (standard deviation) of three independent biological replicates. The single asterisk indicates statistical significance at P < 0.05. The double asterisk indicates statistical significance at P < 0.01.

Supplementary Figure 7 Multiple sequence alignment of *GhCDN-A* gene. DNAMAN8 was used to analyse the multiple sequence alignment of *GhCDN-A* gene. The conserved motif was marked by the black box, the sequence used for VIGS experiment was marked with red box.

Supplementary Figure 8 Gene profiles in glandular and glandless materials. **(A)** Pearson correlation between the glandular and glandless plants. **(B)** Volcano plot showed the overlap of differentially expressed genes, FDR ≤ 0.01 and log_2_|FC| ≥1. **(C)** The KEGG classification of up-regulated genes. **(D)** The KEGG classification of down-regulated genes.

Supplementary Figure 9 The relative expression of key gene. **(A)** The relative expression of *TIFY9* gene in gossypol and gossypol-free materials. **(B)** The relative expression of *GhCDNA* gene in gossypol and gossypol-free materials. **(C)** The relative expression of *GhCDNA* in *GhJAZ1* overexpressed gene materials.
