# Supplementary material for: Genome-wide identification of terpenoid synthase family genes in Gossypium hirsutum and functional dissection of its subfamily cadinene synthase A in gossypol synthesis
Source: Front Plant Sci. 2023 Apr 26;14:1162237. doi: 10.3389/fpls.2023.1162237 (PMC10169749; doi:10.3389/fpls.2023.1162237)
Supplement: Supplementary Figure 1 — Diagram of GhTPSs gene replication. The figure showed the result of collinear analysis of genes in G. hirsutum. The squares on the ring edge represent chromosomes, the names of chromosomes were marked on the top of chromosomes, the length of small squares represents the relative length of chromosomes, and the length was marked on the chromosomes, and the grey lines represented collinear relationship between all the GhTPSs, the blue lines represented collinear relationships between GhCDNs. [file Presentation_1.pptx]

## Slide 1
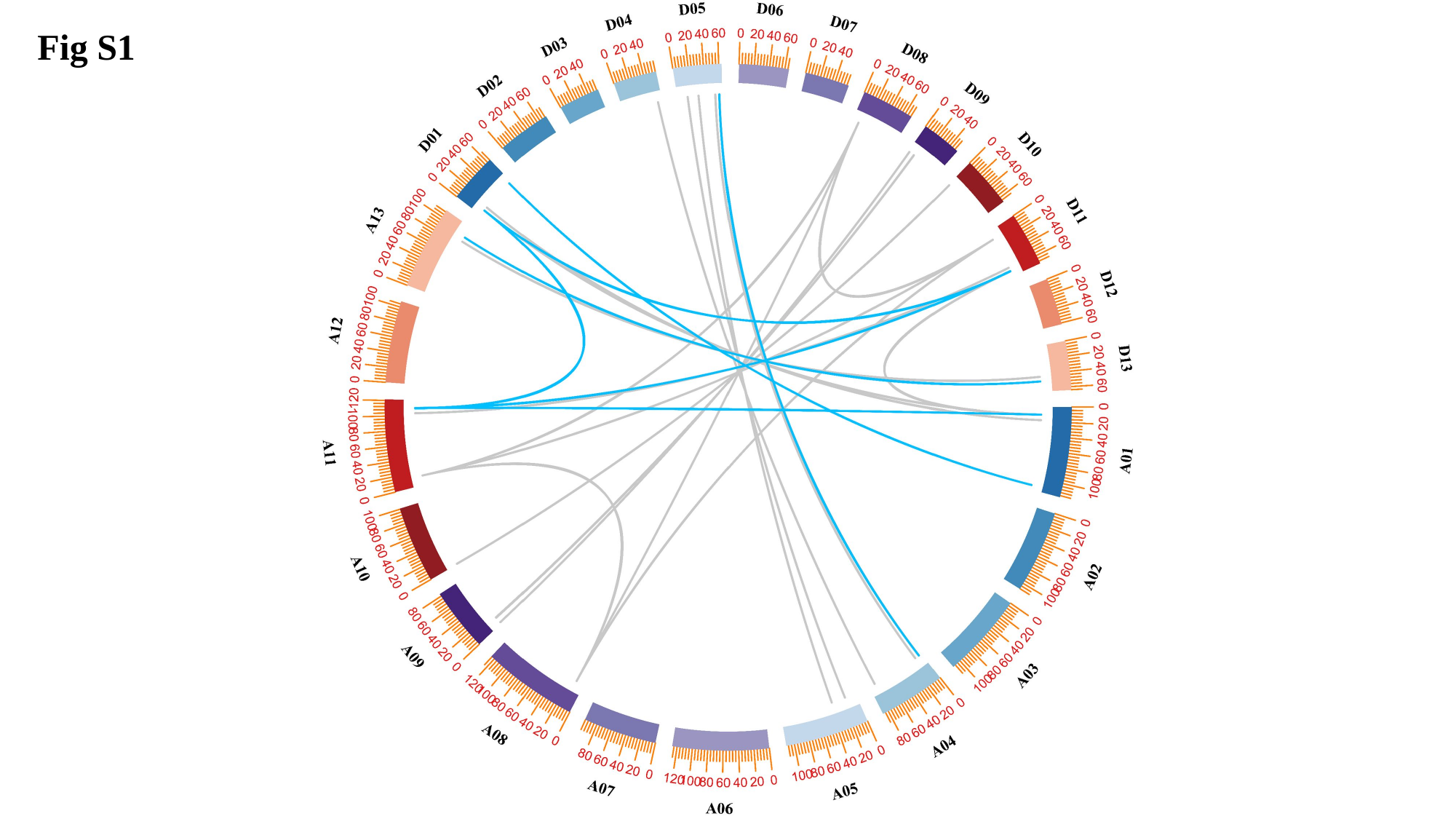

Fig S1

## Slide 2
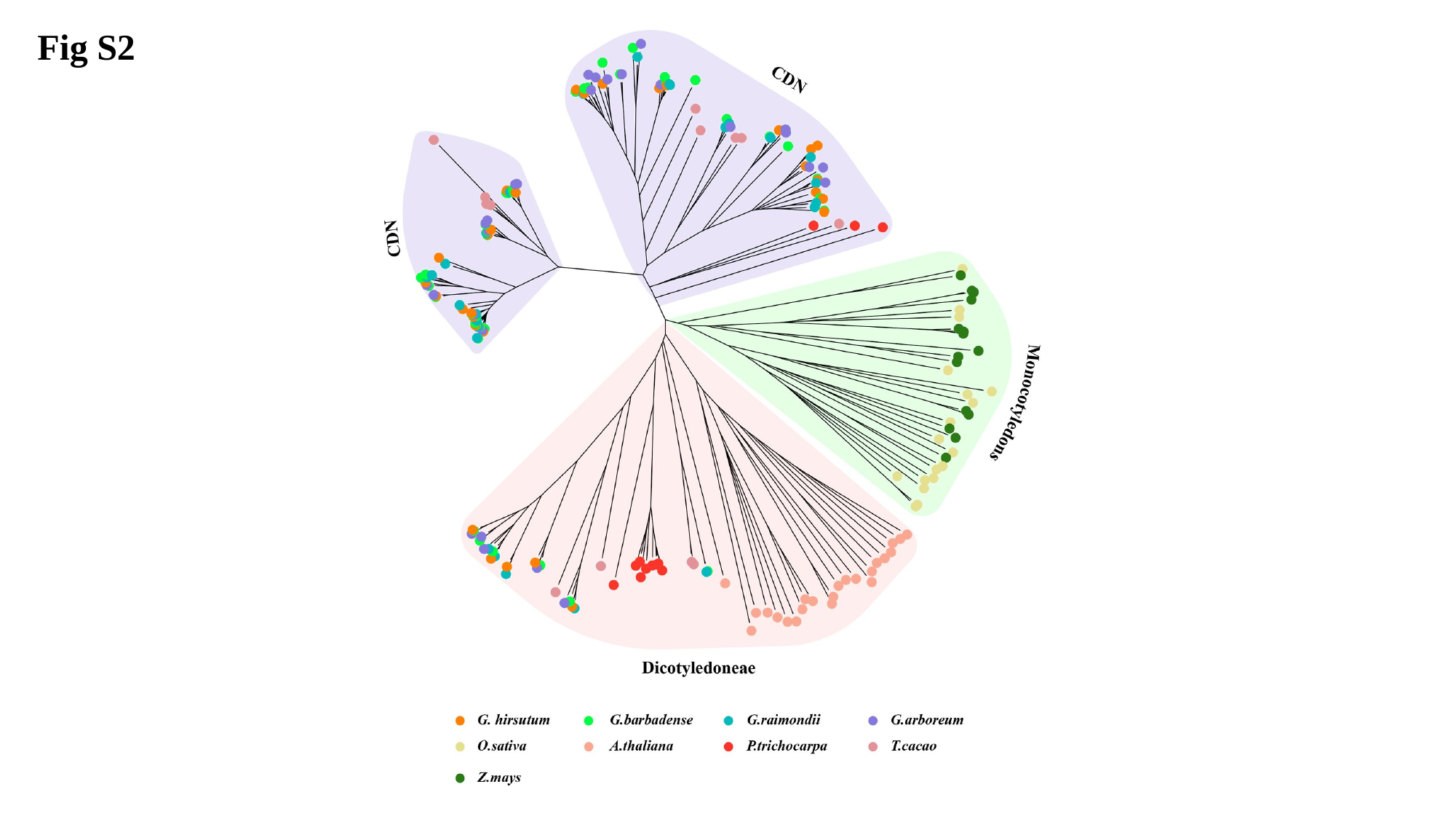

Fig S2

## Slide 3
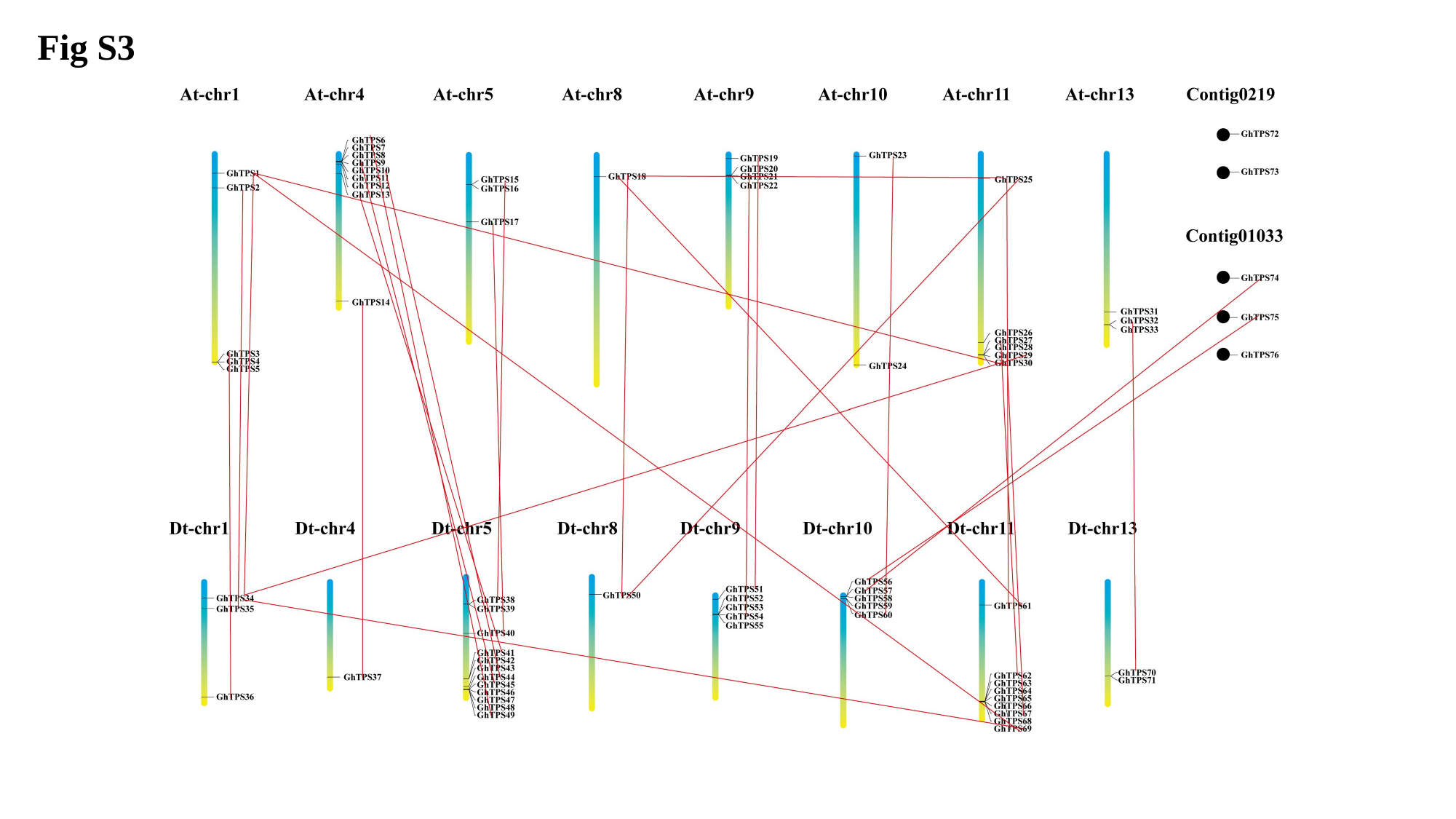

Fig S3

## Slide 4
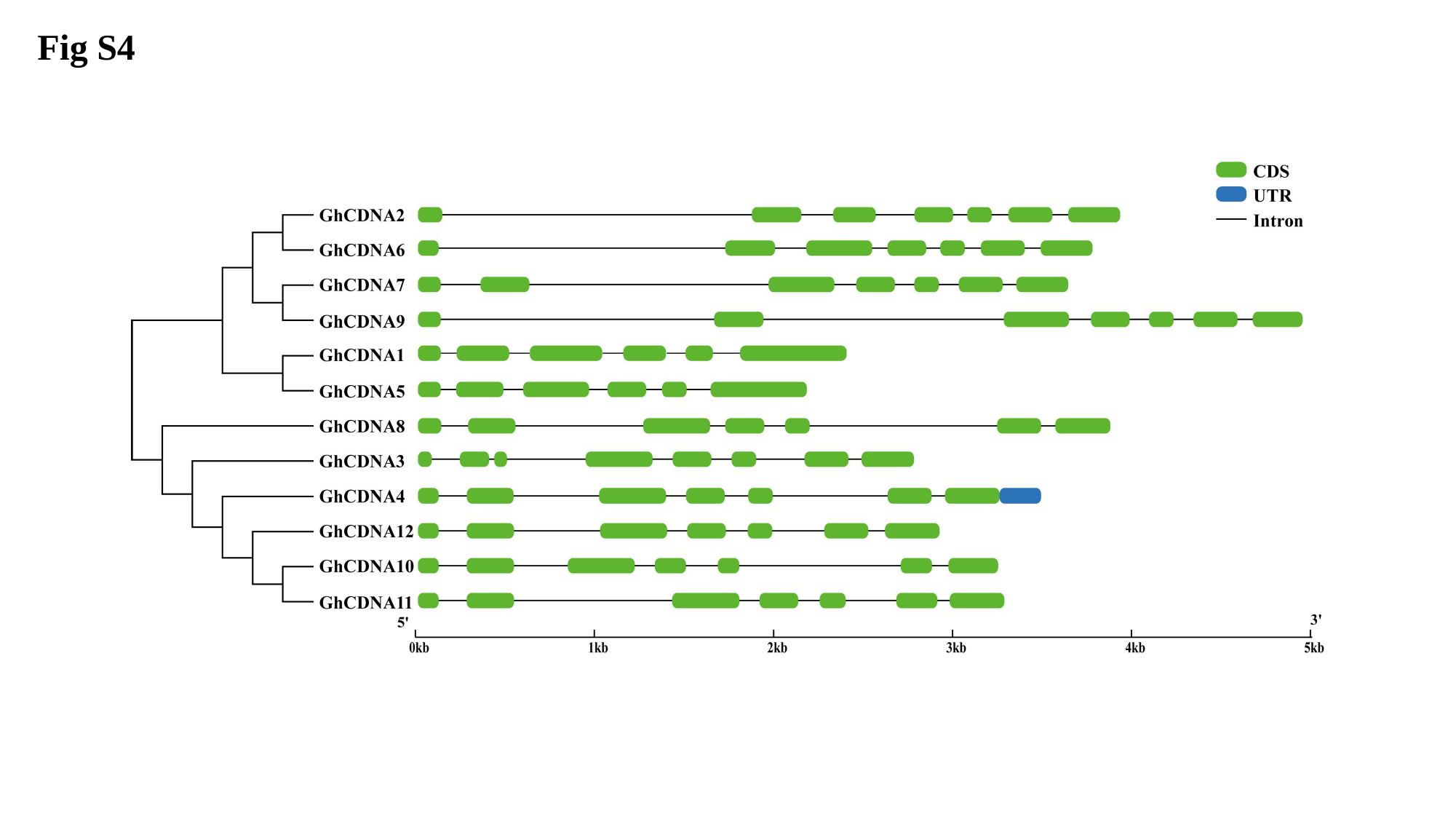

Fig S4

## Slide 5
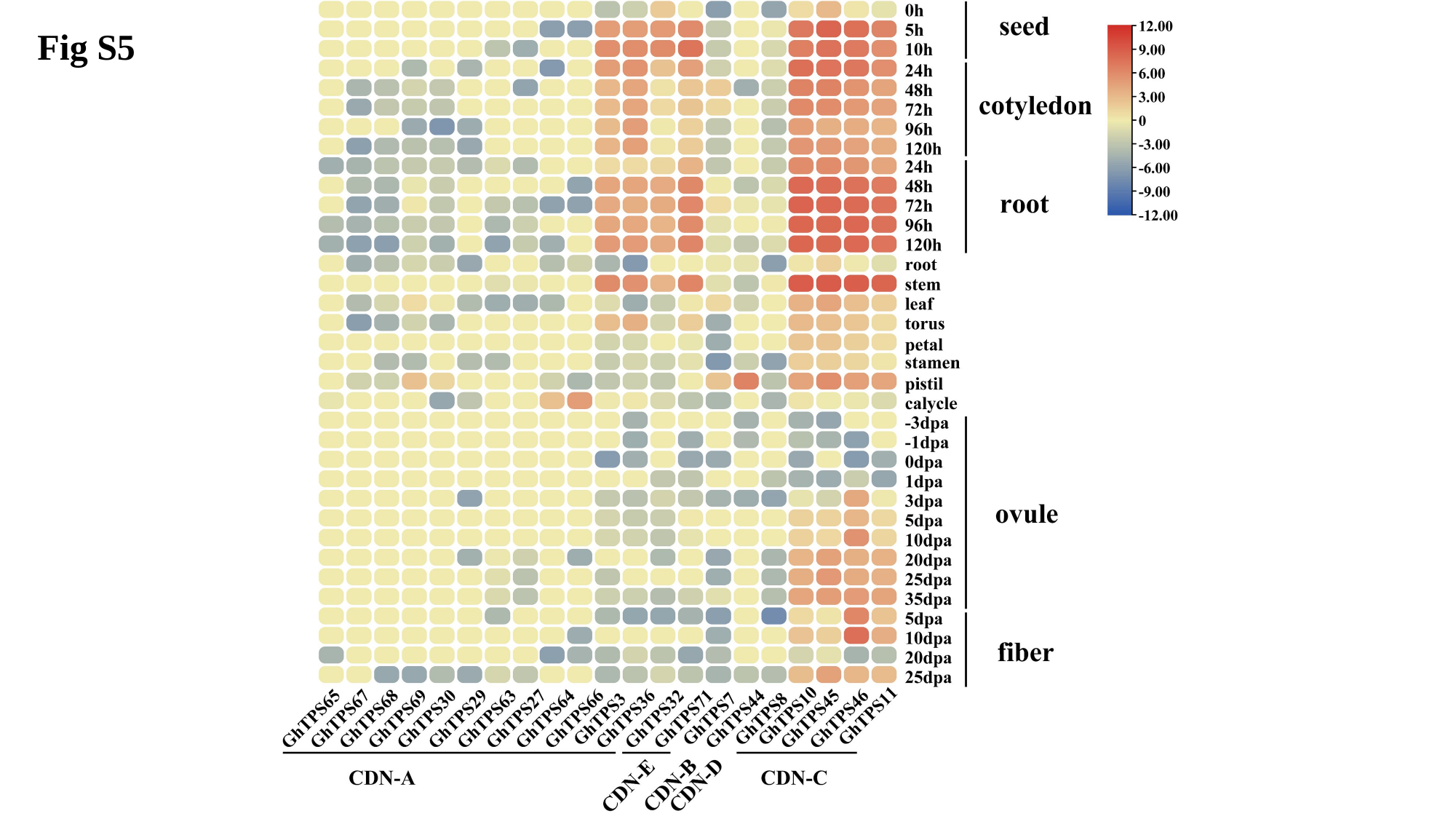

Fig S5

## Slide 6
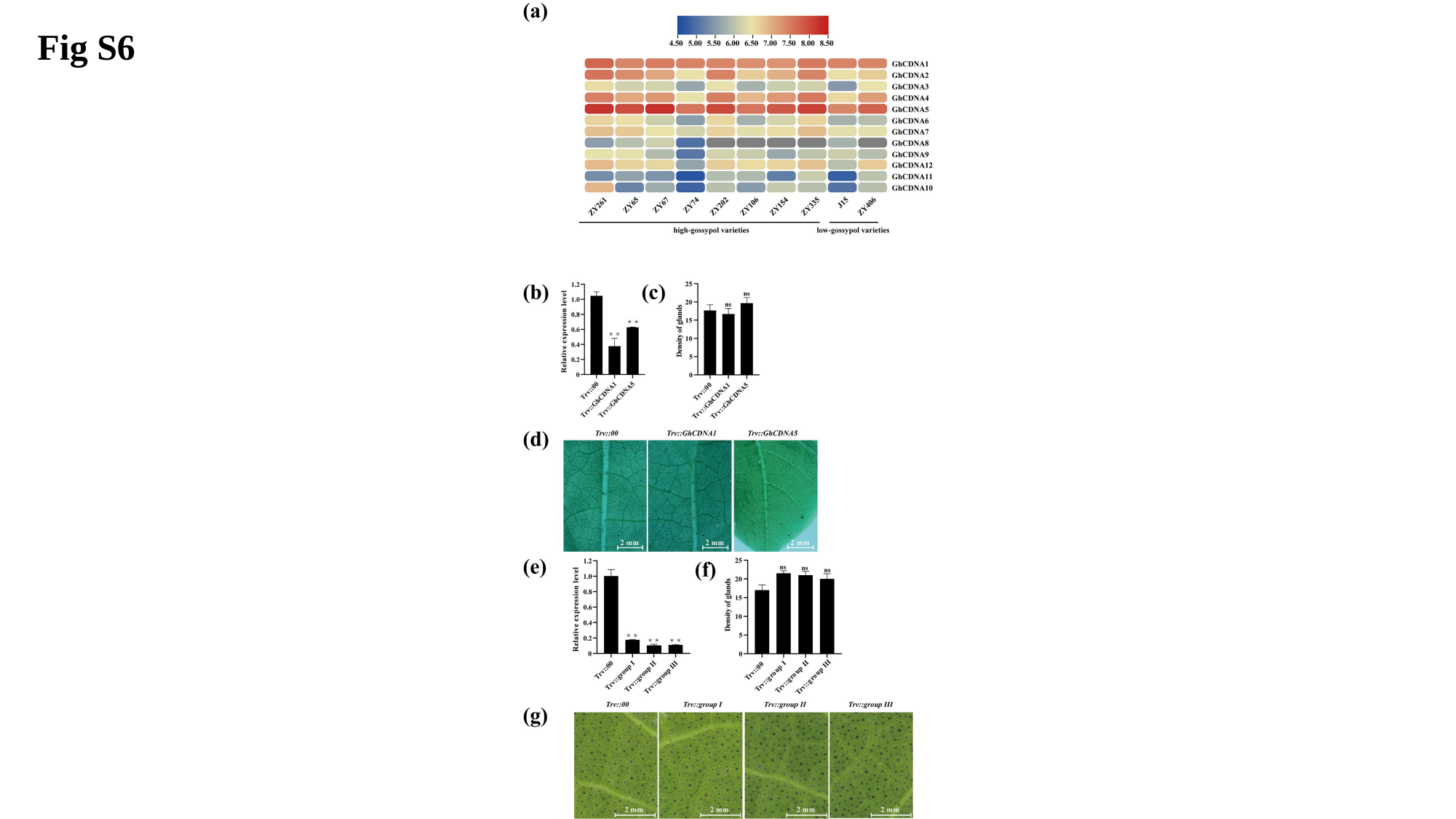

Fig S6

## Slide 7
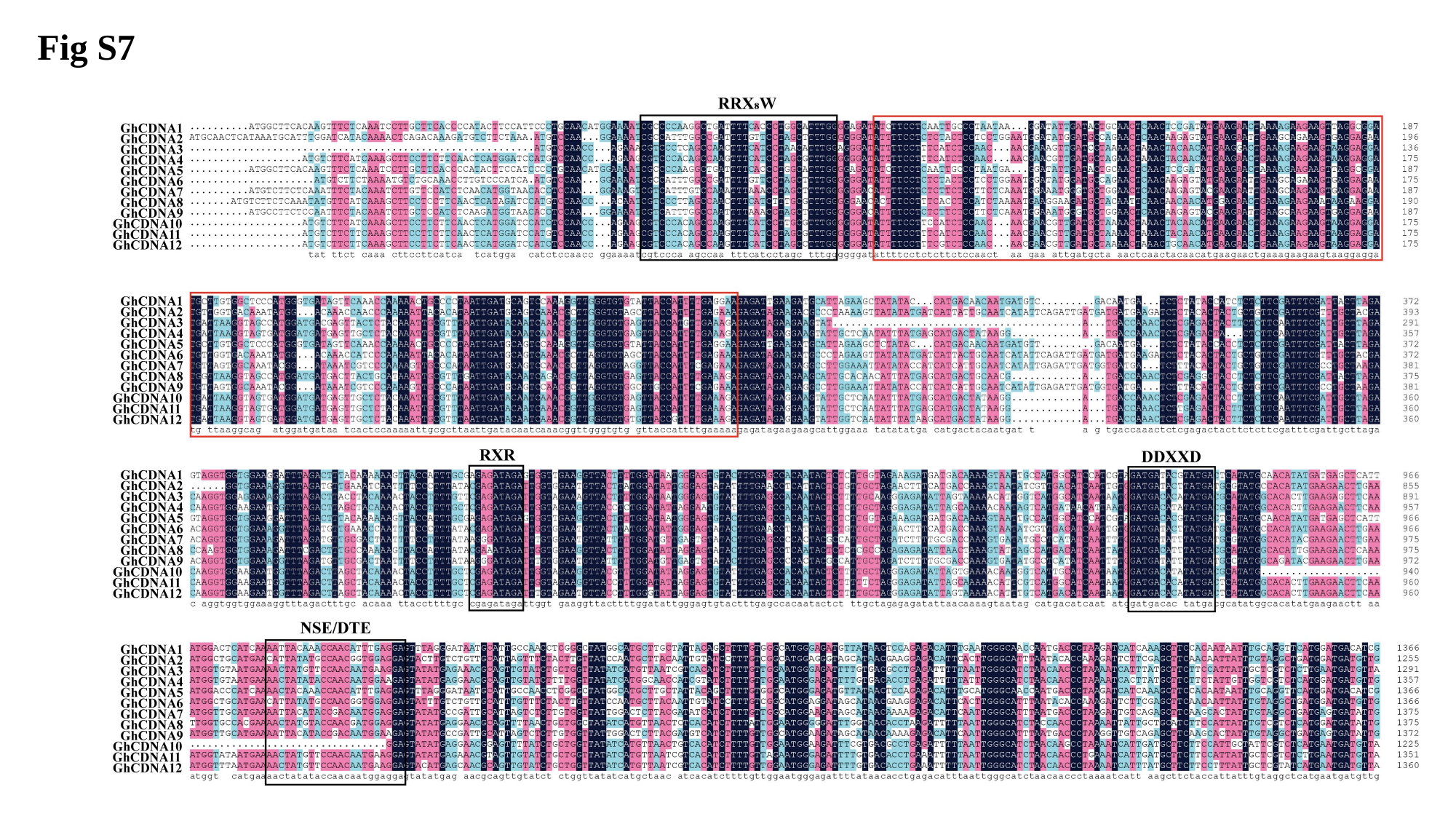

Fig S7

## Slide 8
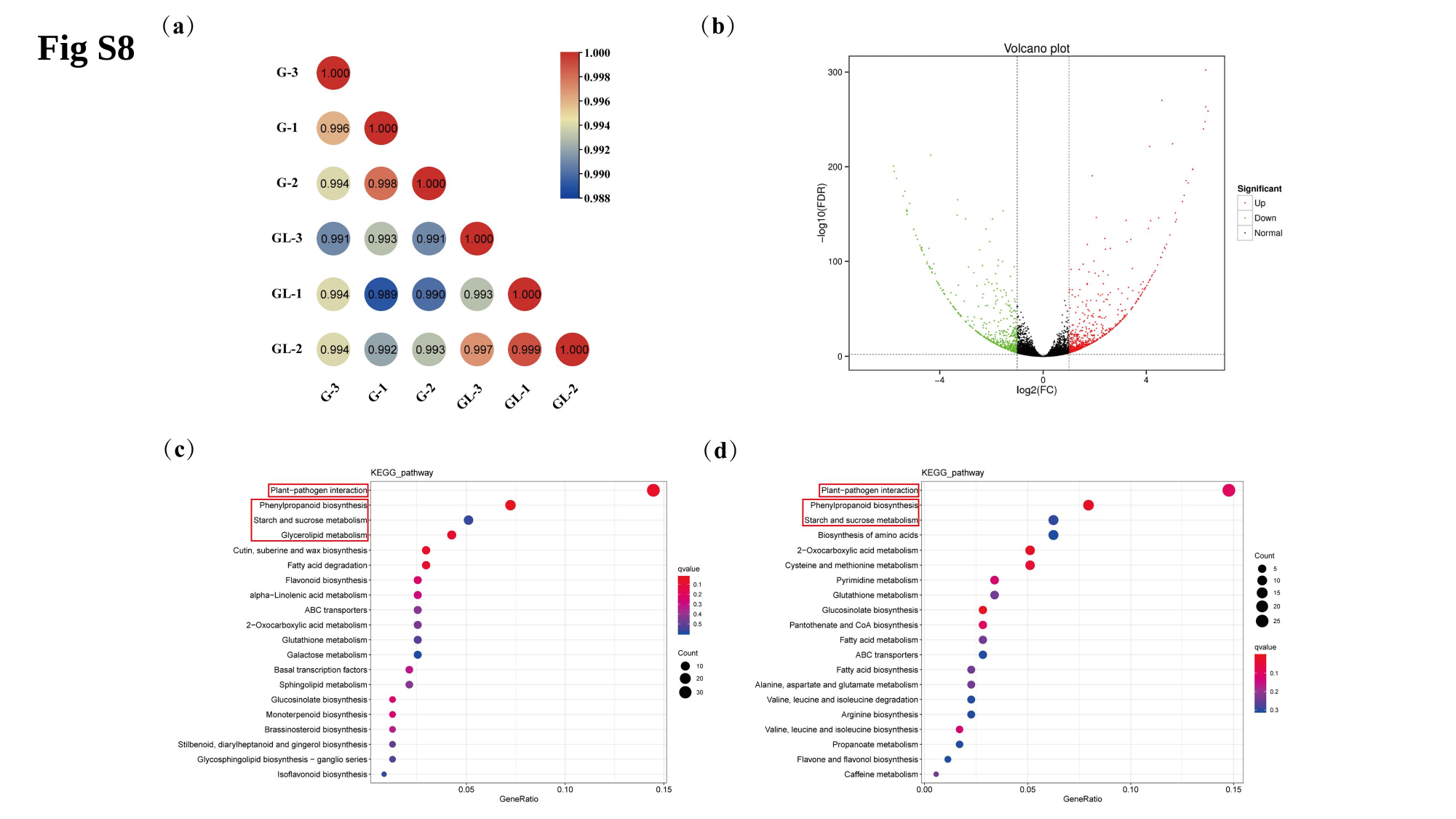

Fig S8

## Slide 9
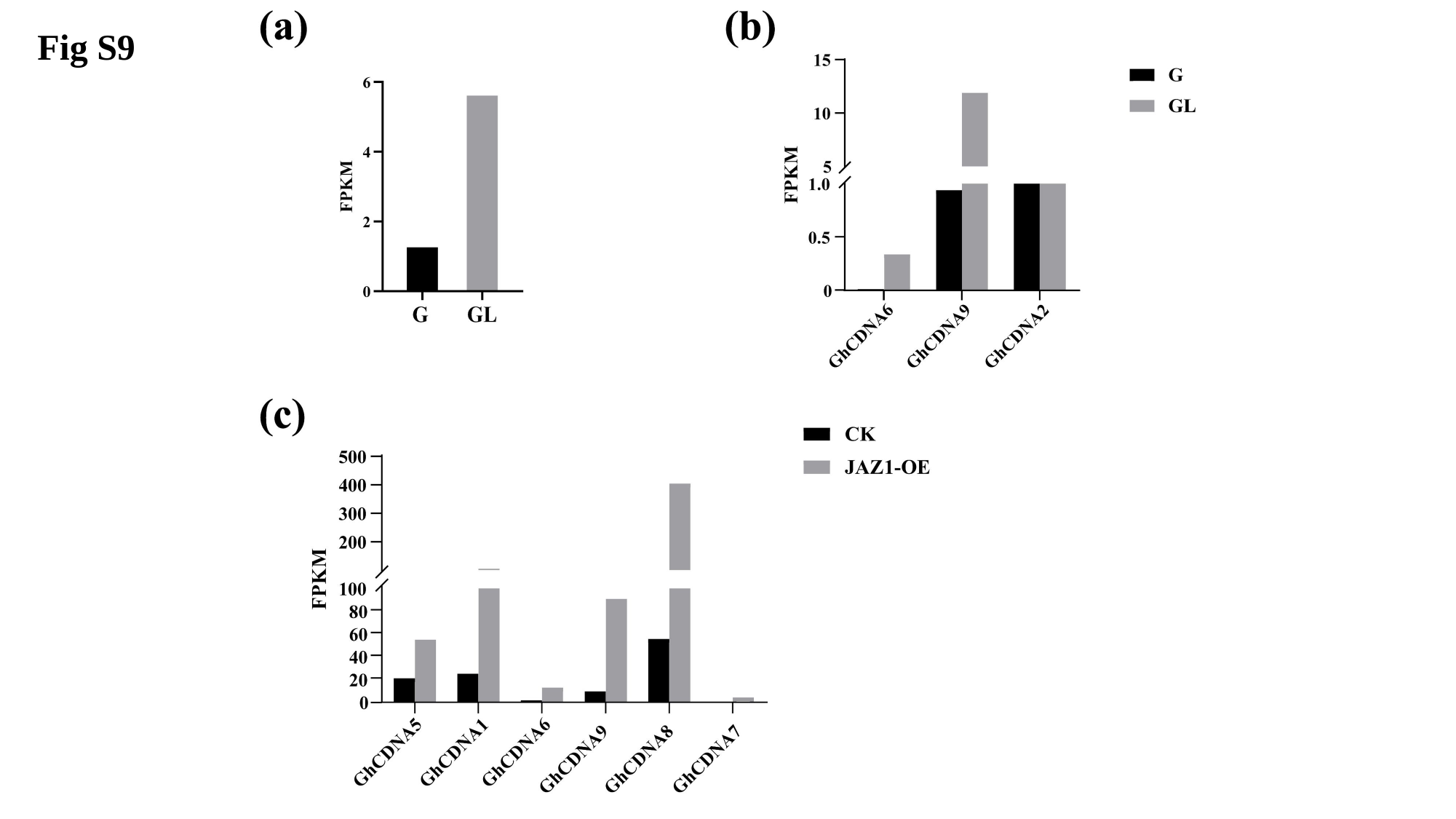

Fig S9
